# Nutritional Pearls: Diet and Rheumatoid Arthritis

**DOI:** 10.31138/mjr.31.3.319

**Published:** 2020-05-15

**Authors:** Panagiotis Athanassiou, Lambros Athanassiou, Ifigenia Kostoglou-Athanassiou

**Affiliations:** 1 Department of Rheumatology, St. Paul’s Hospital, Thessaloniki, Greece; 2 Department of Physiology, Medical School, University of Athens, Greece; 3 Department of Endocrinology, Asclepeion Hospital, Voula, Athens, Greece

**Keywords:** diet, rheumatoid arthritis, fasting, Mediterranean diet, Cretan Mediterranean diet, vegetarian diet, obesity, flavonoids, resveratrol, curcumin

## Abstract

Various studies have investigated the effect of diet on rheumatoid arthritis (RA) as a complimentary treatment along with standard drug therapy. Various types of diet have been investigated. Fasting, the Mediterranean diet, the Cretan Mediterranean diet, vegetarian diet, an anti-inflammatory diet and the use of various specific food substances have been in the focus of research interest. The relationship of obesity with disease activity in RA has also been investigated. A period of fasting followed by Mediterranean diet, the Cretan Mediterranean diet and an anti-inflammatory diet have been found to have a beneficial effect on disease activity in RA. Obesity has been found to be associated with increased disease activity in RA. However, weight loss appears to be related to increased mortality in RA. The use of flavonoids, resveratrol and curcumin may have a beneficial effect in RA. It appears that diet may aid in RA management as a complimentary factor to standard drug treatment.

## INTRODUCTION

The effect of diet as a complimentary factor to standard drug treatment has been extensively investigated in rheumatoid arthritis (RA). Specifically, the effect of fasting,^1^ the Mediterranean diet,^2^ the Cretan Mediterranean diet,^3^ vegetarian diet and specific food substances^4–6^ on disease activity in RA has been investigated in various studies all over the world. Recently, the effect of an anti-inflammatory diet has been investigated in RA.^7,8^ The effect of various diets, and types of behavior such as that leading to obesity and various diet constituents has been investigated in many studies over many years. The aim of this review is to address some aspects of the subject. In particular, the results of various studies performed to assess the effect of diet on RA will be reviewed. Specific subjects will be investigated, such as the effect of fasting, the Mediterranean diet, the Cretan Mediterranean diet and vegetarian diet on RA. The effect of an anti-inflammatory diet on RA will also be discussed. The relationship of obesity with disease activity in RA will be reviewed. The effect of specific food substances on RA will also be reviewed.

## FASTING

In the era immediately before the introduction of biologic agents, the belief was widely held that diet may be a useful adjunct to the usual treatment of RA. Therefore, many studies were performed with diet interventions as a complimentary factor to the treatment of RA. The scientific investigation of the effect of fasting and a lactovegetarian diet on RA was pioneered by Swedish doctors. In a study performed in Sweden more than thirty years ago, Skoldstam et al.^1^ investigated the effect of fasting and lactovegetarian diet on RA. A group of 16 patients with RA underwent a 7–10 day period of fasting followed by a 9-week period on a lactovegetarian diet. A group of 10 RA patients acted as controls and followed their normal diet. After fasting, an improvement was observed in 5/15 patients, while in only 1 of the controls. The fasting patients showed reduced pain, stiffness, consumption of analgesics and several clinical variables. At the conclusion of the lactovegetarian period, only one diet patient showed improvement. In a study performed in Norway^9^ the effect of fasting followed by a year on a vegetarian diet was studied in a single-blind, randomized controlled trial. The subjects all suffering from RA were put in a subtotal fast for 7–10 days followed by a period of a vegan diet for 3.5 months and a lacto-vegetarian diet for the rest of a year. A beneficial effect in RA was observed. In particular, the number of tender and swollen joints decreased, the Ritchie’s articular index improved, the HAQ score improved and erythrocyte sedimentation rate and CRP also improved. It was concluded that the dietary regimen seemed to be a useful supplement to conventional medical treatment of RA. The same authors followed up their study population of RA patients for two years and observed that diet responders had sustained clinical benefit over the period of two years.^10^ The effect of fasting followed by various diets has been investigated further and it was found that fasting followed by vegetarian diets may be useful in the treatment of RA.^11^ Studies were performed to investigate the way that fasting may influence disease activity in RA. In particular, Sundqvist et al.^12,13^ investigated the effect of fasting and a lacto-vegetarian diet on intestinal permeability and disease activity in RA patients. They found that fasting decreased intestinal permeability and ameliorated disease activity in RA. Fraser et al.^14^ investigated the effect of fasting on interleukin-6 (IL-6) and dehydroepiandrosterone sulphate (DHEAS) levels in patients with RA and they found that fasting decreased serum levels of IL-6 by 37% (p<0.03) and improved disease activity. They also found that fasting increased DHEAS levels. They concluded that the fall in IL-6 concentrations may underlie the fall in ESR and CRP in RA patients after a 7-day fast. Fraser et al.^15^ investigated the matter further by measuring the levels of total and free cortisol in RA patients with active disease on a total 72h water fast who were not previously treated with glucocorticoids. They observed a significant increase in total and free cortisol which occurred mainly at night. During the 3-day fast, 24h free and total cortisol concentrations increased markedly. This was due mainly to a marked increase in nocturnal serum cortisol concentrations during fasting compared to the fed state. They concluded that the increase in endogenous cortisol concentrations observed during a short fast may underlie the significant improvement in RA patients observed during longer fast periods. In another study Fraser et al.^16^ investigated the effects of acute starvation in RA patients on mitogen-induced T cell activation and Th1/Th2 cytokine responses. They found decreased CD4+ T cell activation and an increase in the number and function of IL-4 producing Th2 cells. Recent evidence suggests that fasting may affect T lymphocyte generation, function and survival^17^ and thus may affect autoimmunity and immunosenescence.

## MEDITERRANEAN DIET AND THE CRETAN MEDITERRANEAN DIET

The Mediterranean diet is based on the consumption of fruits, cooked vegetables, legumes, olive oil, a small amount of fish, white meat, and a limited amount of red meat and white sugar. The effect of the Mediterranean diet on the prevention of cardiovascular disease has been extensively investigated, and it was observed that it can prevent cardiovascular disease.^18,19^ Skoldstam et al.^3^ investigated the effect of the Cretan Mediterranean diet on disease activity in patients with RA. Patients had active, but well-controlled RA. They adhered to a Cretan Mediterranean diet for 12 weeks, as opposed to a control group who received a standard diet. In the patients with RA who received the Mediterranean diet, a decrease in DAS28 and HAQ was observed, as well as an improvement in physical function and vitality. McKellar et al.^2^ investigated the effect of adherence to the Mediterranean diet in a group of female patients with RA living in a socially deprived area of Glasgow. They observed a beneficial effect of the Mediterranean diet on pain, early morning stiffness, and HAQ. A systematic review performed in 2018 examined the effects of Mediterranean diet in patients with rheumatoid arthritis.^20^ The review concluded that Mediterranean diet has beneficial effects in people living with RA in reducing pain and improving physical function. However, the review concluded that there is insufficient evidence to recommend the widespread use of the Mediterranean diet for the prevention of RA. In a study performed in Sweden based in subjects from the population of the Swedish epidemiological investigation of RA (EIRA),^21^ it was found that adherence to the Mediterranean diet may decrease RA risk. On the contrary, in a study performed in a large population female-only group formed from two large cohorts, the Mediterranean diet was not found to decrease RA risk.^22^ A review assessing the effect of the Mediterranean diet on RA did not find any beneficial effects.^23^ A recent study evaluated the effects of an anti-inflammatory diet on RA.^24^ The diet was based on the principles of a Mediterranean and a vegetarian diet. The food portfolio contained mainly fish, legumes, fruit and vegetables. The study was a single-blinded crossover trial in which 50 patients with RA were randomly assigned to an intervention anti-inflammatory diet or a control diet, the usual Swedish diet, for 10 weeks.^8^ The study showed that the DAS28-ESR decreased significantly during the intervention period and was significantly lower after the intervention than after the control period. It is therefore possible that adherence to the Mediterranean diet with an increased consumption of fatty fish, reduced consumption of sugary drinks and maintenance of a normal body weight may contribute to a reduced risk of RA.^25^

## VEGETARIAN DIET

The effect of a vegetarian diet on rheumatoid arthritis has been investigated in the trial by Kjeldsen-Kragh et al.^9^ In this study, the effect of fasting and a consequent period of vegetarian diet was investigated. The study concluded that this intervention may be beneficial in patients with RA. The same group found a sustained beneficial effect of vegetarian diet on RA patients over a period of two years.^10^ A review of studies on diet intervention in RA concluded that fasting followed by a vegetarian diet is useful for the treatment of RA (*Table 1*).^11^ A review of the use of various types of diet in RA concluded that diet interventions in RA had weight loss as an effect.^26^

**Table 1.** Types of diet which may have a beneficial effect in RA patients as a complimentary factor to standard drug treatment.

**Fasting**
**Mediterranean diet**
**Cretan Mediterranean diet**
**Vegetarian diet**
**Anti-inflammatory diet**

## SUGAR AND RHEUMATOID ARTHRITIS

In a large study performed in the United States, 79,570 women from the Nurses’ Health Study and 107,330 women from the NHS II were followed up.^27^ Information on sugar-sweetened soda consumption was obtained and cases of RA were documented and validated. Women who consumed more than 1 serving of sugar-sweetened soda per day had an increased risk of developing seropositive RA. It has been shown that increased sugar consumption may increase inflammatory stress.^28^

## OBESITY AND RHEUMATOID ARTHRITIS

Obesity is characterized by an inflammatory internal mi-lieu^29^ which leads to increased rates of atherosclerosis. Obesity is accompanied by the release of proinflammatory cytokines by the abdominal fat which characterizes the metabolic syndrome.^30,31^ In a meta-analysis on the relationship of obesity with disease activity in RA, it was found that obesity is accompanied by higher disease activity as assessed by DAS28 in patients with RA, lowers the odds of achieving disease remission in RA, and negatively impacts disease activity and patient-reported outcomes during treatment.^32^ However, obesity was not found to be associated with increased mortality. In another study evaluating 470 patients with RA from the GO-FORWARD and GO-BEYOND randomized clinical trials, it was found that patients with obesity and RA have lower rates of DAS28 remission;^33^ however, similar rates of MRI activity as compared to patients without obesity. The relationship of obesity with RA is complex and characterized by the so-called “obesity paradox”. This paradox refers to the fact that although obesity may be a risk factor for the development of a disease, obese patients appear to have a better prognosis.^34,35^ The obesity paradox most probably reflects the absence of an underlying severe disease. In a very large study in US veterans with RA who were followed up until death or through 2013, it was observed that the highest weight-loss rate was associated with a higher risk of cardiovascular mortality and cancer mortality.^36^ Overweight BMI was found to be protective of cardiovascular mortality. Underweight BMI was associated with markedly increased risk of respiratory mortality. The obesity paradox has been observed in other diseases as well,^37,38^ and may be partly explained by the weight loss accompanying a severe underlying illness.^39–41^ Otherwise stated, an overweight or obese BMI may reflect the absence of significant weight loss due to an underlying illness.^42^

## FLAVONOIDS AND RHEUMATOID ARTHRITIS

Flavonoids are thought to act on the immune system and have immunomodulatory action.^43–45^ Flavonoids are polyphenolic compounds which are found in fruits, vegetables, legumes or cocoa, ie, in food items of plant origin.^46^ Flavonoids may have anti-inflammatory properties.^47^ Studies have shown that flavonoids can inhibit regulatory enzymes or transcription factors important for controlling mediators involved in inflammation. Flavonoids are known to be potent antioxidants, which may attenuate tissue damage or fibrosis.^48,49^ Genistein is an isoflavonoid derived from soy thought to be beneficial in human health.^50^ It is thought to have antioxidant activity and suppressive action on the immune system reducing the number of T cells.^51^ Genistein has been shown to modulate experimental autoimmune encephalomyelitis induced in mice.^51^ Genistein has been found to have a beneficial effect in patients with RA. Genistein has been shown to attenuate collagen-induced arthritis in mice,^52^ and has a potential for the treatment of rheumatoid arthritis.^53,54^

## RESVERATROL AND RHEUMATOID ARTHRITIS

Resveratrol is a polyphenol, a substance found in the skin of grapes, blueberries, raspberries, mulberries and peanuts.^55^ Resveratrol is a phytoalexin, a substance produced by a plant in response to injury or when it is under attack by pathogens,^56^ such as bacteria or fungi. Resveratrol is thought to have multiple beneficial health effects.^57–59^ Resveratrol has antioxidant properties.^57^ Resveratrol has been shown to ameliorate experimental arthritis in rats.^60^ In a rat model of arthritis resveratrol was found to inhibit angiogenesis and to ameliorate experimentally induced arthritis^5^. In a randomized controlled clinical trial, resveratrol was added to the conventional treatment of RA in 50 patients, while 50 patients received only the conventional RA treatment and acted as controls.^61^ The DAS28 activity index and serum inflammation markers decreased in the resveratrol treated group. The use of polyphenols in the management of autoimmune disorders is currently discussed.^62^

## CURCUMIN AND RHEUMATOID ARTHRITIS

Curcumin is a yellow pigment found in the spice turmeric, and a main functional constituent of the rhizomes of Curcuma longa.^63^ Thus, curcumin is a derivative of the root of a plant belonging to the Zingiber species.^64^ Curcumin is a hydrophobic polyphenol which is a strong antioxidant and anti-inflammatory agent with multiple effects. Three major curcuminoids exist, namely, curcumin, demethoxycurcumin, and bis-demethoxycurcumin. Curcuminoids have been shown to lower CRP.^65^ Curcumin constitutes 1%–5% of turmeric preparations and has a molecular weight of 368.37 and the molecular formula C_21_H_20_O_6_.^66^ Curcumin has long been used as a food (eg, in the popular Indian curry), a colouring agent, and in traditional medicine.^67^ Curcumin has been applied successfully in the treatment of arthritis.^68^ Curcumin has been also used for the treatment of RA.^69^ The use of curcumin has been also proposed for the management of both periodontitis and RA and for the prevention of the latter.^4^ The use of curcumin in therapeutics has many problems, as it is unstable at physiological pH, it has low solubility in water, and it is rapidly metabolized.^63^ Therefore, several novel preparations have been tested to overcome these problems. A novel curcumin preparation and a hydrogenated curcuminoid formulation have been tested for the treatment of RA.^70,71^ Co-encapsulation of curcumin and resveratrol in lipid-core nanoparticles has been proposed as a method to improve bioavailability of curcumin, stability of both substances, and their antioxidant capacity.^72–74^

## CONCLUSION

Fasting followed by a period of Mediterranean diet or the Cretan Mediterranean diet or vegetarian diet as well as an anti-inflammatory diet have been shown to have a beneficial effect as a complimentary factor in the treatment of RA. Obesity has been shown to be related to increased disease activity in RA, however not with increased mortality. The use of flavonoids, resveratrol and curcumin may have beneficial effects in RA patients.
